# Task matters: an investigation on the effect of different secondary tasks on dual-task gait in older adults

**DOI:** 10.1186/s12877-021-02464-8

**Published:** 2021-09-25

**Authors:** Hui-Ting Goh, Miranda Pearce, Asha Vas

**Affiliations:** 1 School of Physical Therapy-Dallas, Texas Woman’s University, Dallas, TX USA; 2 School of Occupational Therapy-Dallas, Texas Woman’s University, Dallas, TX USA

## Abstract

**Background:**

Dual-task gait performance declines as humans age, leading to increased fall risk among older adults. It is unclear whether different secondary cognitive tasks mediate age-related decline in dual-task gait. This study aimed to examine how type and difficulty level of the secondary cognitive tasks differentially affect dual-task gait in older adults.

**Methods:**

Twenty young and twenty older adults participated in this single-session study. We employed four different types of secondary tasks and each consisted of two difficulty levels, yielding eight different dual-task conditions. The dual-task conditions included walking and 1) counting backward by 3 s or by 7 s; 2) remembering a 5-item or 7-item lists; 3) responding to a simple or choice reaction time tasks; 4) generating words from single or alternated categories. Gait speed and cognitive task performance under single- and dual-task conditions were used to compute dual-task cost (DTC, %) with a greater DTC indicating a worse performance.

**Results:**

A significant three-way interaction was found for the gait speed DTC (*p* = .04). Increased difficulty in the reaction time task significantly increased gait speed DTC for older adults (*p* = .01) but not for young adults (*p* = .90). In contrast, increased difficulty level in the counting backward task significantly increased gait speed DTC for young adults (*p* = .03) but not for older adults (*p* = .85). Both groups responded similarly to the increased task difficulty in the other two tasks.

**Conclusions:**

Older adults demonstrated a different response to dual-task challenges than young adults. Aging might have different impacts on various cognitive domains and result in distinctive dual-task gait interference patterns.

## Background

Approximately 1 in every 4 U.S. residents older than 65 years reports falling each year [1]. The estimated medical cost associated with falls is nearly $50 billion per year [2]. Previous efforts have identified several risk factors for falls, including female gender and older age; neither of which is modifiable [3–5]. There is an urgent need to identify the modifiable factors of falls such that targeted interventions can be developed. Dynamic functional activities, such as walking, and cognitive function are the two critical modifiable predictors of falls [6]. Specifically, executive function domains including attention, inhibitory control, working memory, and cognitive flexibility are essential for safe walking [7]. Dual-task walking is a daily activity that simultaneously challenges both dynamic balance and executive function. It is highly associated with falls [8] and recommended to be included in fall risk screening [9, 10].

Dual-task gait assessment often involves walking while performing another cognitive task. Various cognitive tasks have been used in dual-task gait research including serial subtraction, generating words, responding to a stimulus, etc. [11–13]. Despite the variations in protocol, studies consistently reported a reduced gait speed during dual-task walking, which is attributed to limited attentional resource [8, 13]. According to the single-attentional resource model, attention is a *single* fixed-capacity resource and dual-tasking leads to performance deterioration because the attentional capacity is exceeded [14]. However, the single-attentional model does not provide a satisfactory explanation when the type of secondary task mediates dual-task performance [15–19]. Beauchet et al. reported that older adults showed a marked increase in gait variability during dual-task walking with an arithmetic task, but not with a verbal fluency task [16]. This task-dependency might be better explained by the Wickens’s multiple-resource attention model [20]. In this model, the attentional system consists of multiple resources, each with its own capacity. Dual-task interference only occurs when both tasks tax the *same* resource pool [20, 21]. Based on this model, each attentional resource may show different degrees of susceptibility to brain injury or aging and lead to task-dependent dual-task interference in different populations [15, 18]. However, the task-dependency in dual-task walking has been limited examined in geriatric research [22, 23]. Al-Yahya and colleagues performed a meta-analysis to compare the effect of different cognitive task domains on dual-task gait in healthy and neurological populations. The authors concluded that cognitive tasks requiring internal processing such as mental tracking and verbal fluency tended to lead to greater dual-task interference than tasks involving external interfering factors such as reaction time [24]. The findings from this meta-analysis signify the role of cognitive task type in dual-task gait.

Another explanation for the task-dependency is task difficulty, which has been shown to affect dual-task gait performance [22, 25]. Lovden et al. used an n-back task to study how levels of difficulty influence dual-task walking. In the n-back task, participants needed to respond whenever they heard a digit that was repeated one (1-back, easiest) to four (4-back, most difficult) positions back in the series. In their study, older adults’ gait performance was differently impacted by various difficulty levels of the n-back task [25]. Therein, previous studies that found a task-dependency in dual-task walking could also be explained by the different difficulty imposed by various tasks. However, it is unfeasible to compare difficulty levels across different tasks (e.g. arithmetic vs. verbal fluency) because they represent distinct skills and are measured on different scales. It is unclear if older adults are more susceptible to a specific type or a more difficult secondary task. The purpose of this study was to examine how type and difficulty level of the secondary cognitive tasks differentially affect dual-task gait in older adults by comparing older adults’ responses to that of young adults. We hypothesized that compared to young adults, older adults would show a greater dual-task gait deterioration as difficulty level increased. Furthermore, the magnitude of gait deterioration would depend on the task type.

## Methods

### Participants

A priori power analysis was conducted to determine the minimum sample size required. We used a moderate effect size of 0.25 (f), a power of .80, α-level at .05, and a correlation among the repeated measures of 0.3. The power analysis indicated that a minimum of 32 participants was required. We considered a 20% attrition rate and determined a total sample size of 40 (20 per group). The inclusion criteria were: able to walk independently and continuously for at least 30 s with or without an assistive device; normal or corrected vision and hearing; and able to understand and speak English. Additional inclusion criteria were an age of 65–90 years for the older adults and 18–45 years for the young adults. Participants were excluded if they had a history of neurological disorders, significant musculoskeletal issues in the lower body that would interfere with walking, cognitive impairment (Montreal Cognitive Assessment (MoCA) < 24/30), or unstable medical issues. All participants signed a written informed consent and a local institutional review board approved the study.

### Ethical approval

This study was reviewed and approved by a local institutional review board. The study was conducted according to the Declaration of Helsinki. All participants signed a written informed consent prior to participation.

### Procedures

The study was a single-session repeated measure design. At the beginning of the session, participants’ demographic and medical information was recorded followed by assessment of cognitive and balance function. General cognitive function was assessed using the MoCA [26] and executive function was assessed with the Trail-Making Test (TMT A and B) [27]. Participants also performed the Digit Span Forward and Digit Span Backward tests to evaluate their working memory [28]. Balance and self-efficacy for balance were assessed with the Four-Stage Balance Test [29] and the International Falls Efficacy Scale [30].

We then instructed the participants to walk for 30 s at their preferred speed on a pre-marked oval path. A 4-ft by 26-ft Zeno Walkway mat (Protokinetics, USA) was placed along the straight length of the path to capture gait parameters. The single-task walking trial was repeated three times with a 30-s rest between trials. Next, they performed one of the four cognitive tasks at either the easy or difficult level in standing for 30 s. We administered the cognitive tasks in standing instead of sitting to ensure that participants assumed the same posture between single- and dual-task conditions. The changes in cognitive task performance could then be attributed to the addition of walking but not the changes in posture. The cognitive task performance was recorded by a wireless microphone. The order of the four tasks was pseudo-randomized within each group. Half of the participants within each group started with the easy level while the other half started with the difficult level. After performing the cognitive task in standing once, the participants performed the same cognitive task while walking simultaneously for three 30-s trials. Participants were instructed to put their priority on the cognitive task because previous studies have shown that task prioritization significantly affect task performance [31, 32]. It would be difficult compare the task performance if no explicit instruction was given because each individual might adopt different strategies. To ensure that we could confidently compare the effects of various tasks on gait performance, we instructed participants to prioritize the cognitive task during dual-task walking. After completing three trials, the participants had a one-minute seated rest break before performing the same cognitive task at the other difficulty level, first under the single-task standing condition then under the dual-task walking condition. Participants repeated the same procedure for all four secondary tasks at each difficulty level, yielding eight dual-task walking conditions.

#### Secondary tasks

We utilized four different types of secondary cognitive tasks: counting backward, list recall, reaction time, and verbal fluency. These tasks are commonly used in dual-task gait assessment and each engages specific cognitive process [12, 13, 24]. For the counting backward task, participants were given a number ranging from 300 to 900 and instructed to count backward by 3 (easy) or by 7 (difficult) for 30 s and a greater number of correct responses suggested a better performance. Counting backward is thought to engage numerical processing skills and is associated with activation in the bilateral prefrontal cortices, left posterior inferior parietal lobule, and left supplementary motor area [33, 34]. Counting task is considered a type of mental tracking tasks requiring holding information in the working memory and performing mental manipulation [24]. Mental tracking tasks are shown to lead to high levels of interference during dual-task walking [24].

For the list recall task, participants listened to a pre-recorded list of items (easy 5 items or difficulty 7 items) for 30 s and recalled the items after 30 s. The rate of correct recalls (in %) was recorded. This task is purported to rely on episodic memory encoding and retrieval processes and has been associated with the activation of bilateral prefrontal cortices [35, 36]. This task requires holding information in the working memory without manipulation [24]. Al-Yahya et al. reported that the working memory task resulted in significant dual-task interference [24].

When performing the reaction time task, participants listened to a pre-recorded 30-s audio track consisting of twelve audio stimuli with various inter-stimulus-intervals. For the easy level, all twelve stimuli were high pitch tones (1000 Hz) and participants needed to respond by saying “High” as soon as they heard the signals. For the difficult level, six high pitch tones (1000 Hz) were randomly mixed with six low pitch tones (500 Hz) and participants were instructed to respond as quickly and accurately as possible by saying “High” or “Low” accordingly. The duration between the onsets of the sound signal and the verbal response was recorded; a shorter duration indicated better performance. Reaction time tasks index information processing speed and vigilance [31, 37, 38] and have been associated with the activation of the primary motor cortex, premotor cortex [39], and dorsolateral prefrontal cortex [40]. In the meta-analysis conducted by Al-Yahya et al., the reaction time tasks were found to result in the least interference to gait speed, though it was still statistically significant [24].

For the verbal fluency task, participants were asked to generate as many words as possible from a predetermined category (e.g. fruit) for 30 s. The easy level of this task included just one category while the difficult level required alternating between two categories (e.g. fruit-animal-fruit-animal). A greater number of appropriate words generated indicated better performance. Verbal fluency tasks have been shown to evoke activation in the frontal network [41] and temporal lobe [42]. Interestingly, verbal fluency tasks have been found to result in similar magnitude of interference as mental tracking tasks but they are not associated with age while the effect of mental tracking task was age-dependent [24]. The easy level of this task engages semantic knowledge and retrieval processes [43]. The difficult level of this task additionally engages cognitive flexibility and set-shifting because participants needed to switch between categories [44].

### Outcomes and analysis

Our primary outcomes were dual-task cost (DTC) in gait speed and cognitive task performance. We calculated DTC using the following formula: $DTC\ \left(\%\right)=\frac{Dual- task\ performance- Single- task\ performance}{Single- task\ performance}x\ 100\%$. A greater DTC implicates greater performance deterioration under dual-task conditions. Normality and outliers were checked to see if parametric analysis assumptions were met. Independent t-tests or Chi-square tests were performed to compare the demographics between groups. If a demographic outcome was significantly different between groups and theoretically confounds the primary outcomes, it was then treated as a covariate in the primary analyses. We conducted 2 (groups) × 4 (tasks) × 2 (difficulty levels) repeated-measure ANOVAs or ANCOVAs on the DTC of gait speed and cognitive task performance. Post-hoc Bonferroni adjusted pairwise comparisons were conducted if a main effect or interaction was found. Dual-tasking can lead to different interference patterns based on task challenges and prioritization [45]. We also descriptively analyzed the interference patterns by contrasting DTC in gait speed to DTC in cognitive task performance. All analyses were completed in SPSS v.25 with *p* < .05 was set as the significance.

## Results

Twenty older adults (mean age 72.5 ± 5.5 years) and twenty young adults (mean age 26.3 ± 4.5 years) participated in this study (Table 1). Two older adults reported a fall in the last 12 months while none of young adults fell. Compared to young adults, older adults were significantly older, shorter in body height, and spent fewer years in formal education. Older adults had significantly worse performance than young adults in the Digit Span Backward Test, TMT, and single-leg stance balance (the fourth item on the 4-Stage Balance Test). Older adults also walked slower and reported a greater concern about falling than young adults.

**Table 1:** Demographic information (mean (SD) or N (%))

	Young Adults (*N* = 20)	Older Adults (*N* = 20)	*p*
Gender (N, %)	Female = 12 (60%)	Female = 16 (80%)	.17
	Male = 8 (40%)	Male = 4 (20%)	
Age (years)	26.30 (4.49)	72.50 (5.47)	**.00**
Body Weight (kg)	71.99 ± 13.60	72.73 ± 13.21	.80
Body Height (cm)	169.22 ± 11.20	162.50 ± 7.92	**.04**
First language	English = 16 (80%)	English = 17 (85%)	.83
	Spanish = 2 (10%)	Spanish = 1 (5%)	
	Other = 2 (10%)	Other = 2 (10%)	
Years of Education (years)	19.68 ± 2.30	15.10 ± 3.49	**.00**
MoCA (max = 30)	27.00 ± 2.58	26.50 ± 2.57	.55
Digit span forward (max = 16)	10.90 ± 2.83	9.65 ± 2.80	.16
Digit span backward (max = 14)	8.70 ± 3.44	6.05 ± 2.67	**.01**
TMT-A (seconds)	14.09 ± 3.99	33.23 ± 15.29	**.00**
TMB-B (seconds)	34.65 ± 12.00	91.20 ± 39.39	**.00**
4-stage balance tests (seconds, max = 10)			
1	10.00 ± 0.00	10.00 ± 0.00	1.0
2	10.00 ± 0.00	10.00 ± 0.00	1.0
3	10.00 ± 0.00	8.70 ± 2.71	.29
4	10.00 ± 0.00	6.90 ± 3.10	**.01**
Walking speed (m/s)	1.41 ± 0.10	1.15 ± 0.18	**.00**
FES-I (16–64)	16.30 ± 0.57	25.95 ± 9.15	**.00**
Fall in the last 12 months	Yes = 0 (0%)	Yes = 2 (10%)	.15
	No = 20 (100%)	No = 18 (90%)	

*MoCA Montreal Cognitive Assessment, *TMT* Trail Making Test, *FES-I* Fall Efficacy Scale-International

**Fig. 1:**
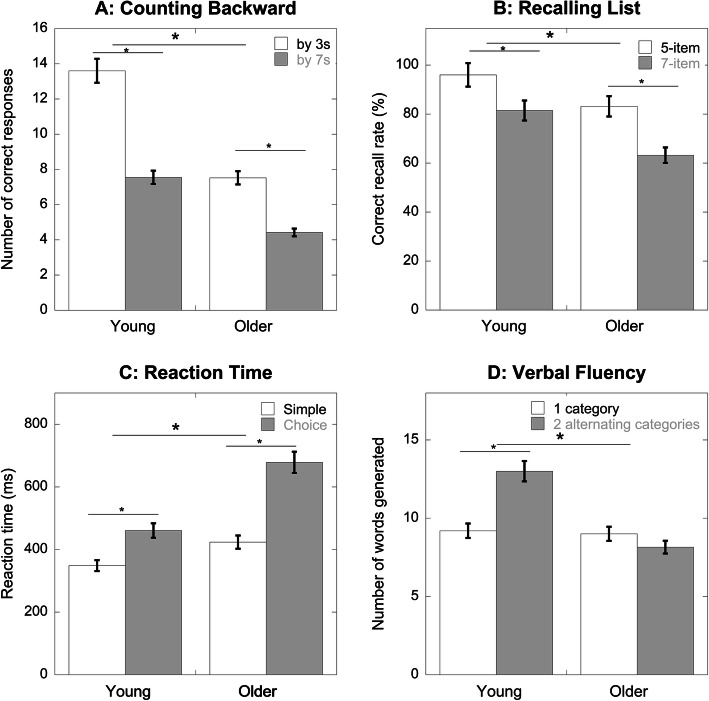
Performance on the cognitive tasks (mean, SE) under single-task standing condition across two difficulty levels between young (*N* = 20) and older (*N* = 20) adults. Asterisks indicate significant differences between difficulty levels or between age groups

We first compared participants’ single-task (standing) cognitive performance across the two difficulty levels as a manipulation check (Fig. 1). Participants generated fewer correct responses when counting backward by 7 s compared to by 3 s (F_1,37_ = 125.52, *p* < .01). Overall, older adults showed a significantly lower number of correct responses than young adults (F_1,37_ = 13.55, *p* < .01), as well as a greater decline in counting performance as the task difficulty increased (F_1,37_ = 12.97, *p* < .01) (Fig. 1A). For the list recall task, participants’ performance was worse with the 7-item list compared to the 5-item list (F_1,37_ = 40.91, *p* < .01) and older adults had a lower correct recall rate compared to young adults (F_1,37_ = 14.45, *p* < .01) (Fig. 1B). Both groups responded slower to the choice reaction time task compared to the simple reaction time task (F_1,37_ = 104.30, *p* < .01). Older adults responded slower compared to young adults (F_1,37_ = 22.21, *p* < .01) and demonstrated a greater decline than young adults when task difficulty increased (F_1,37_ = 15.82, *p* < .01) (Fig. 1C). Older adults, compared to young adults, generated fewer words for the verbal fluency task (F_1,37_ = 15.77, p < .01). There was also a significant group by difficulty interaction (F_1,37_ = 27.03, *p* < .01). Older adults generated a similar number of words between the easy (single category) and difficult (alternating categories) levels (*p* = .15). In contrast, young adults generated significantly more words under the difficult condition than the easy condition (*p* < .01). Overall, the difficulty manipulation was effective for all tasks except the verbal fluency task.

**Fig. 2:**
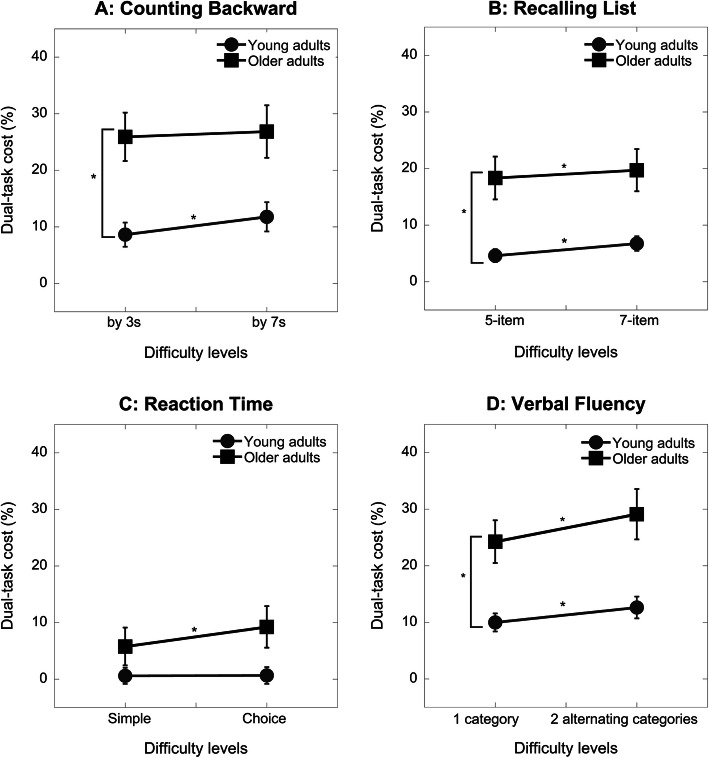
Dual-task cost (mean, SE) in gait speed under different task conditions (**A**: Counting backward; **B**: Recalling list; **C**: Reaction time; **D**: Verbal fluency) between young (*N* = 20) and older (*N* = 20) adults. Asterisks indicate significant differences between difficulty levels or between age groups

Because groups were different in years of education and levels of education has been shown to influence dual-task gait performance [46], we first performed a 2 groups × 4 tasks × 2 difficulty levels repeated measures ANCOVA with years of education served as a covariate. The years of education did not have significant effects on the gait speed DTC. We thereby analyzed DTC in gait speed using a 2 groups × 4 tasks × 2 difficulty levels repeated measure ANOVA and found a significant 3-way interaction (F(3,105) = 2.85, *p* = .04) (Fig. 2). We subsequently performed four separate 2 groups × 2 difficulty levels repeated measure ANOVAs for each task. Older adults showed a greater gait speed DTC (by 3 s mean ± SD = 25.92% ± 19.11%; by 7 s mean ± SD = 26.85% ± 20.21%) than young adults (by 3 s = 8.64% ± 9.56%; by 7 s = 11.79% ± 11.55%) when walking and counting backward (F_1,37_ = 11.08, *p* < .01). The difficulty level (*p* = .08) and group by difficulty level interaction (*p* = .14) were not significant (Fig. 2A). Recalling the 7-item lists resulted in a greater gait speed DTC for both groups as confirmed by a significant difficulty level effect (F_1,37_ = 8.86, *p* = .01) and non-significant group by level interaction (*p* = .53). The older adults showed a greater gait speed DTC (5-item = 18.34% ± 16.48%; 7-item = 19.72% ± 16.25%) than the young adults (5-item = 4.62% ± 5.21%; 7-item = 6.75% ± 5.76%) for the recall task (F_1, 37_ = 12.19, *p* < .01) (Fig. 2B). There was a significant group by difficulty level interaction (F_1,37_ = 6.57, *p* = .01) for the reaction time task (Fig. 2C). Increased difficulty level in the reaction time task did not change the gait speed DTC for the young adults (simple = 0.60% ± 6.40%; choice = 0.67% ± 6.61%) (*p* = .71) but significantly increased the gait speed DTC for the older adults (simple = 5.78% ± 14.94%; choice = 9.24% ± 16.40%) (*p* < .01). DTC in gait speed was significantly greater when participants walked and generated words for alternating categories than single category (F_1,37_ = 16.24, *p* < .01). Older adults showed a greater DTC (1-category = 24.26% ± 16.86%; 2-alternating categories = 29.12% ± 19.90%) than young adults (1-category = 9.99% ± 7.13%; 2-alternating categories = 12.64% ± 8.62%) (F_1,37_ = 12.27, *p* < .01). The group by level interaction was not significant for the verbal fluency task (*p* = 0.24) (Fig. 2D).

**Fig. 3:**
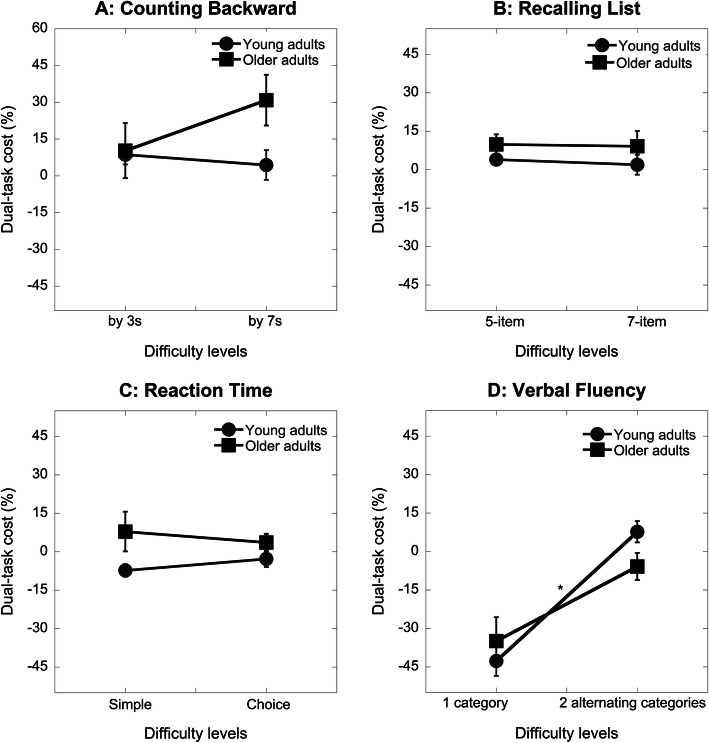
Dual-task cost (mean, SE) in cognitive tasks under different task conditions (**A**: Counting backward; **B**: Recalling list; **C**: Reaction time; **D**: Verbal fluency) between young (*N* = 20) and older (*N* = 20) adults. The asterisk indicate a significant difference between the two difficulty levels of the verbal fluency task

Although we instructed participants to prioritize the cognitive task during dual-task walking, they may have switched their priority, especially under challenged conditions [31, 32, 47]. Further, dual-tasking could result in performance deterioration in both tasks when the demand is high [45]. We further analyzed participants’ DTC in the cognitive task performance with a 2 × 4 × 2 repeated measures ANCOVA with years of education being a covariate. Unlike gait speed DTC, cognitive task DTC was significantly mediated by the years of education. After controlling for years of education, there was a significant task by level interaction (F_3,96_ = 3.17, *p* = .04) and group effect (F_1,37_ = 5.00, *p* = .03). The task by group and level by group interactions were not significant. A post-hoc Wilcoxon signed rank test with an adjusted *p*-value of 0.01 was performed for each task to examine the effect of difficulty within each task. Increased task difficulty did not result in significant differences in cognitive DTC for the counting backward task (*p* = .32, Fig. 3A), list recall task (*p* = .79, Fig. 3B), and reaction time task (*p* = .26, Fig. 3C), but significantly increased cognitive DTC for the verbal fluency task (*p* < .00, Fig. 3D).

**Fig. 4:**
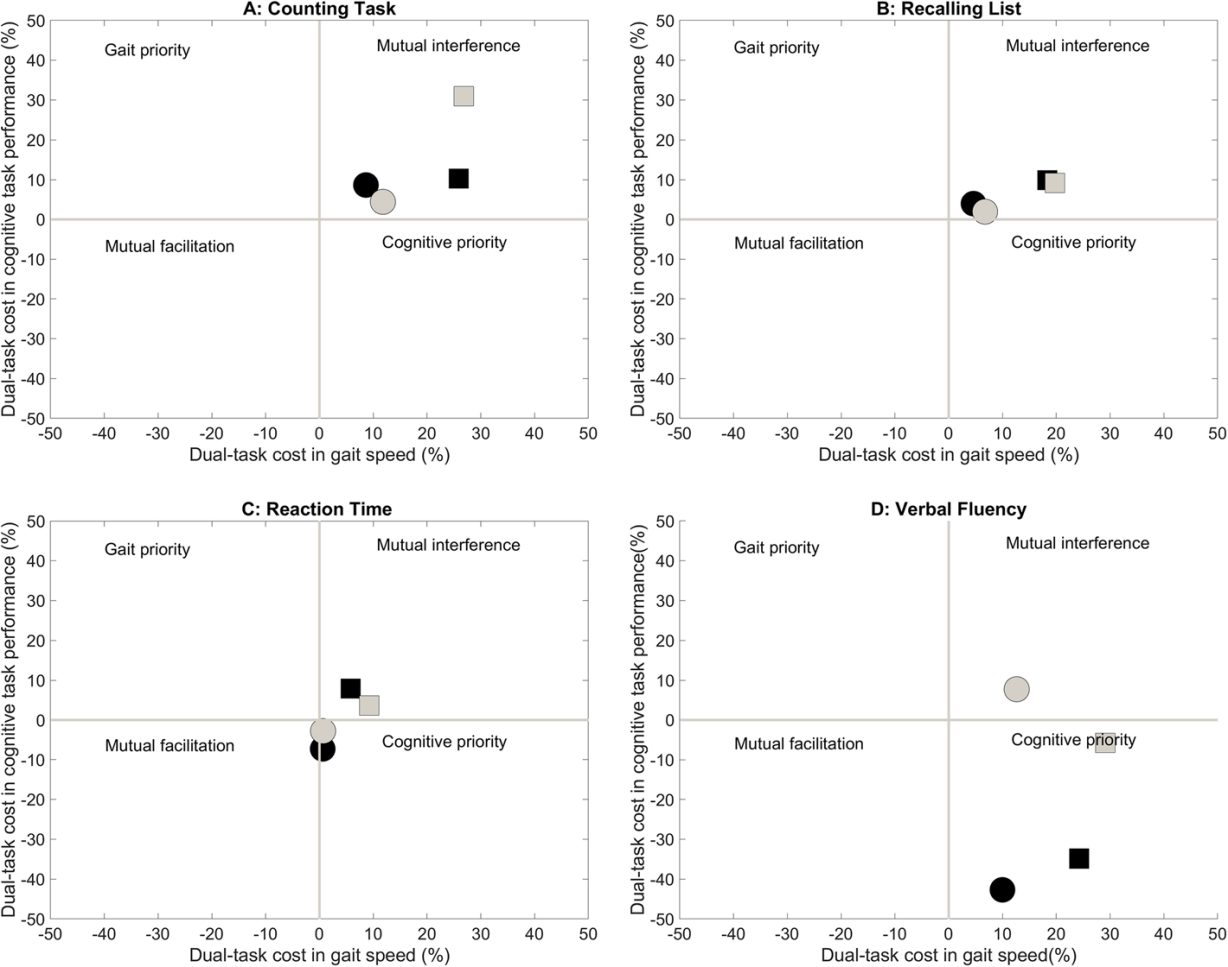
Dual-task interference pattern under four different task conditions (**A**: Counting backward; **B**: Recalling list; **C**: Reaction time; **D**: Verbal fluency) for the young adults (circles) and older adults (squares). Black symbols represent the easy level of the task; gray symbols represent the difficult level of the task

Fig. 4 illustrates the dual-task interference patterns based on the conceptual framework proposed by Plummer et al. [45] Both groups showed mutual interference when they walked and counted backward simultaneously (Fig. 4A). A similar mutual interference pattern was observed when older adults performed list recall task while walking, but young adults showed nearly no interference on the cognitive task performance (Fig. 4B). Young adults also showed no interference in gait and slight facilitation in cognitive task when they walked and performed the reaction time task. In contrast, older adults demonstrated slight mutual interference (Fig. 4C). Both groups demonstrated cognitive task facilitation with the easy verbal fluency task. However, the difficult verbal fluency task led to a mutual interference pattern for younger adults and a cognitive task priority pattern for older adults (Fig. 4D).

## Discussion

Our results showed that older adults, compared to young adults, not only were more susceptible to dual-task interference, but also responded differently to the demands imposed by different dual-task conditions. Our study demonstrated a unique combined effect of secondary task type and difficulty on dual-task gait in older adults. Our findings suggest that the impact of aging on dual-task gait might be domain specific. This domain-specificity should be taken into consideration for the assessment and intervention of dual-task walking in older adults.

### Effects of secondary task type

Similar to previous reports [16, 23, 48], we found that the type of the secondary task modulated the magnitude of dual-task gait decline in older adults. Counting backward led to the greatest interference for older adults as evidenced by the high DTC for both cognitive task and gait speed (Fig. 4A). The verbal fluency task created similar gait speed DTC but lower cognitive DTC than the counting backward task (Fig. 4D). This finding is consistent with a previous report that found a greater DTC in walking while counting backward than walking while reciting alternate letters [49]. Our findings also echo Al-Yahya et al.’s meta-analysis in which the authors found that both mental tracking tasks (i.e. counting backward) and verbal fluency tasks resulted in significant dual-task interference but only the interference imposed by the mental tracking tasks was associated with age [24]. It appears that tasks requiring mental tracking might be more susceptible to aging. Kahlauoi et al. reported that older adults performed similarly to young adults on verbal fluency tasks but showed a reduced prefrontal cortex activation [50]. This reduced prefrontal activation may explain the increase in gait DTC because prefrontal activation is critical for successful dual-task gait in older adults [51]. Older adults probably allocated greater resources to the cognitive task as instructed to achieve a low cognitive DTC but inevitably compromised their gait performance.

List recalling resulted in performance deterioration in both gait and cognitive task performance for older adults but minimal interference for young adults (Fig. 4B). The task engages working memory which is known to decline with aging [52] and therefore resulted in a greater interference for older adults. Older adults showed the least interference when walking with a reaction time task compared to other types of task. Interestingly, young adults exhibited a slightly facilitated cognitive task performance (Fig. 4C); they responded quicker to the audio stimulus under the dual-task condition. This finding is consistent with a previous study in which young adults were found to have better cognitive task performance under dual-task condition [53], probably by promoting a more automatic task processing or increased arousal level [54].

### Effects of secondary task difficulty

Manipulating the difficulty level for each cognitive task led to a few interesting observations. For most conditions, increased task difficulty resulted in an increased gait speed DTC for both groups. However, increased task difficulty in the counting backward task (from by 3 s to by 7 s) did not yield a significant difficulty level effect. A closer inspection of the data revealed that the gait speed DTC increased for younger adults (8.6 to 11.8%) but remained constant for older adults (26.6 to 26.9%) (Fig. 2A). In contrast, older adults exhibited a greater increase in cognitive DTC for the counting performance (12.5 to 30.9%) than young adults (Fig. 3A). It appears that older adults adopted a gait priority strategy, despite being instructed otherwise, when the counting became challenging. Previous studies that compared the effects of counting backward by 3 s versus by 7 s on dual-task gait reported a mixed result [47, 55, 56]. We asked participants to count backward from 3-digit numbers while others often did not specify the numbers used [55, 56]. We also instructed participants to prioritize the counting task, which has been shown to impact DTC [31, 47]. These methodological differences might account for the mixed findings. Increased difficulty in the reaction time task, from simple to choice, also resulted in a different response between groups. Young adults showed minimal changes in gait speed DTC, but older adults showed a significant increase (Fig. 2C). Compared to simple, the choice reaction time task additionally engages response selection processes [57, 58] and inhibition of the alternative responses [37]. Adding response selection demand to the task significantly impacted older adults but not young adults.

Our manipulation of task difficulty was effective for most tasks, except the verbal fluency task (Fig. 1D). Two explanations could account for the contradictory finding in the verbal fluency task. First, generating words for two alternating categories may not be more difficult than one category. On the other hand, the familiarity with the categories could affect the performance. The category used for the easy single-task condition was ‘farm animals’ which may not be familiar for some participants, especially young adults. We compared verbal fluency task performance under the dual-task condition and found that participants generated fewer words under the alternating categories condition (mean (SD) = 10.04 (1.83)) than one category condition (mean (SD) = 12.03 (2.53)). Therefore, the contradictory finding in the verbal fluency task was likely resulted from familiarity with the category. Participants generated fewer words for the ‘farm animals’ category under single-task (standing) conditions than the other categories used under dual-task conditions. This resulted in a negative cognitive DTC for the easy level of the verbal fluency task (Fig. 3D). Interestingly, older adults performed worse than young adults on all cognitive tasks but they generated a comparable number of words in the ‘farm animals’ category. This unexpected finding suggests that difficulty levels of the verbal fluency task might also depend on familiarity with the category [50]. Compared to single category, alternating between two categories significantly deteriorated gait speed because it required task switching which might additionally tax the executive function [44]. Notably, older adults showed a similar increase in gait DTC as young adults when task difficulty increased (Fig. 2D). In addition to the verbal fluency task, older adults also showed comparable increase in gait speed DTC as young adults with the increased difficulty of the list recalling task (Fig. 2B).

Taken together, the effect of task difficulty on age-related dual-task gait seems to be task-dependent. Compared to young adults, older adults appeared to respond differently to increased difficulty for the least demanding (i.e. the reaction time) and the most demanding (i.e. counting backward) tasks. A direct comparison of the processing demands imposed by different tasks is challenging. However, by examining the DTC (a unit-less measure), a comparison of task demands might be feasible. The counting backward task led to the greatest DTC while the reaction time led to the least. It could be that older adults had reached the maximal resource capacity with the counting backward task and subsequently showed minimal changes in gait speed DTC as task difficulty increased. In contrast, young adults might require minimal to no resources to perform the reaction time task and thus had no changes in DTC as task difficulty increased. Older adults have been shown to utilize more neural resources than young adults to perform simple tasks [59] and they are likely to reach the ceiling effect under high demand conditions.

Our findings also suggest that the task difficulty might be relative for each age group. The same increment in task difficulty could result in different magnitudes of performance deterioration for young and older adults [60]. It appears that older adults were more susceptible to the increased difficulty in the counting backward and reaction time tasks because they showed a greater task performance decline as difficulty level increased under the single-task condition (see Fig. 1). The unequal responses to difficulty increment might contribute to the differences observed in gait speed DTC. Using an equivalent relative difficulty such as previously done in Qu et al. [48] might be useful to further examine how task difficulty impacts dual-task gait performance in older adults.

### Limitations

Our sample consisted of relatively high functioning older adults with only two participants reporting a history of falls.. Further, our sample size was small and consisted of mostly females. Our sample characteristics might limit the generalizability of our findings. We only investigated the cognitive tasks that are most commonly used in dual-task gait assessment. Previous research has reported detrimental effects of visual-spatial tasks (e.g. checking a visual target) [51] and motor tasks (e.g. holding a tray) [17, 61] on dual-task gait. We purposefully excluded these tasks because they share common input (vision) and output (motor) modalities with gait control, and thus may not delineate the effects of specific cognitive domains [62]. It is unclear if similar interference patterns would be observed if the secondary task was a sensory or motor task. Furthermore, a greater variation in task difficulty may reveal the ‘turning-point’ of dual-task gait decline [25, 54]. Lovden and colleagues investigated the effect of an n-back task with 4 levels of nominal difficulty on dual-task gait and identified different turning-points for young and older adults [25]. We recommend future research to extend our findings by including more difficulty levels to examine how task difficulty mediates dual-task gait. Further, task difficulty level was determined nominally in our study. The same increment in difficulty (e.g. from by 3 s to by 7 s) might produce different levels of *relative* difficulty for each individual or age group. We used task performance to index the difficulty which clearly suggested that older adults were differentially impacted by the increase of difficulty levels. We recommend future research examine both nominal and relative difficulty levels for example probing participant’s perception of task difficulty to further examine the impacts of task difficulty. Similarly, we only examined straight walking while walking complexity (e.g. obstacle negotiation) is also an important mediator for dual-task gait performance [22, 55]. We instructed participants to prioritize the cognitive task. It would be worthwhile to examine how task type and difficulty affect individual prioritization without explicit instruction. Lastly, it remains to be determined if the task-dependency reported here predicts different levels of fall risk among older adults.

## Conclusions

In conclusion, our study found a unique task dependency in age-related dual-task gait decline. Older adults’ susceptibility to dual-task interference is mediated by both type and difficulty of the secondary cognitive task. Therapists are recommended to take into considerations of these two factors to assess and treat dual-task gait decline in older adults.

## Data Availability

The datasets used and/or analyzed during the current study are available from the corresponding author on reasonable request.

## Abbreviations

MoCA: Montreal Cognitive Assessment
DTC: Dual-task cost
TMT: Trail Making Test
